# The Use of Coping Strategies for Everyday Challenges by University Students: Brazil‐Finland Cross‐National Study

**DOI:** 10.1111/sjop.70013

**Published:** 2025-08-06

**Authors:** Livia Oliveira dos Santos, Lucas Arrais de Campos, Adrielly dos Santos, Timo Peltomäki, Tella Lantta, Jaakko Varpula, João Maroco, Juliana Alvares Duarte Bonini Campos

**Affiliations:** ^1^ School of Pharmaceutical Sciences São Paulo State University (UNESP) São Paulo Brazil; ^2^ Faculty of Health Sciences, Institute of Dentistry University of Eastern Finland Kuopio Finland; ^3^ Faculty of Medicine and Health Technology Tampere University Tampere Finland; ^4^ Tampere University Hospital Tampere Finland; ^5^ Department of Oral and Maxillofacial Diseases Kuopio University Kuopio Finland; ^6^ Department of Nursing Science University of Turku Turku Finland; ^7^ Centre for Forensic Behavioural Sciences Swinburne University of Technology Melbourne Australia; ^8^ Department of Biomedical, Metabolic and Neuroscience University of Modena and Reggio Emilia Reggio Emilia Italy; ^9^ Lusófona University Lisbon Portugal

**Keywords:** coping, cross‐national comparison, mental health, university students

## Abstract

The coping pattern of individuals who experience different cultures is influenced by different worldviews and ways of dealing with problems. This study aimed to estimate the psychometric properties of the BriefCOPE inventory and to compare coping strategies between Brazilian and Finnish university students. The study also aimed to identify how individual characteristics relate to coping strategies and explore the interconnections among strategies within the student samples from both countries. This is a cross‐sectional observational study. Data was collected in Brazil using paper‐and‐pencil (*n* = 398, female = 66.6%; mean age = 21.0, SD = 2.2 years) and in Finland using an online survey (*n* = 165, female = 67.3% mean age = 26.9, SD = 7.2 years) during 2023 and 2024. A demographic questionnaire and the BriefCOPE Inventory were used. The fit of BriefCOPE to the samples was estimated using confirmatory factor analysis. Prevalences of coping strategies commonly used by students were calculated using a 95% confidence interval (95% CI). Multiple logistic regression models were developed, and the odds ratio (OR) was estimated considering each sample and its characteristics. Network analysis was carried out to identify the interconnection among coping strategies. The BriefCOPE Inventory presented adequate psychometric properties in both samples after refinement. Brazilian students showed a higher prevalence of using “Planning” and “Self‐Distraction” strategies, while Finnish students showed a more uniform and balanced use of all coping strategies. In Brazil, students who reported having some type of mental health care had a greater probability of using adaptive strategies (“Active Coping”: OR = 3.51). In Finland, individual characteristics seem to have little effect on the choice of coping strategies. For both samples, “Planning” was the main strategy in maintaining networks. Finnish students have a larger and diverse repertoire to face everyday problems and better manage psychosocial demands compared to Brazilian students. Expanding students' coping repertoire can be important in preventing the development of symptoms associated with mental disorders in response to stress.

**Summary**

- After examining the psychometric properties of BriefCOPE, factor models differed between Finland and Brazil, indicating a lack of configural invariance.
- Cross‐national findings revealed that Finnish students adopt a broader range of coping strategies and manage psychosocial demands more effectively, whereas Brazilian students tend to adopt polarized strategies, focused on either solving or avoiding problems.
- Differences in coping strategies between Brazilian and Finnish students may reflect both individual coping repertoires and the distinct nature and complexity of everyday challenges they face.
- Considering the positive correlation between mental health maintenance and adaptive coping strategies, expanding students' coping repertoire may be important for preventing the development of symptoms related to mental disorders in response to everyday challenges.

## 1 Introduction

Coping strategies are different ways of dealing with stress and everyday challenges based on thoughts and attitudes, which can be compiled and studied using the BriefCOPE Inventory (Carver 1997). This instrument allows for the distinction of behavioral, emotional, and cognitive resources adopted in situations that require the mobilization of a repertoire to deal with internal and external demands of the relationship between the individual and the environment (Lazarus and Folkman 1984). Furthermore, from a theoretical point of view, BriefCOPE strategies can be grouped into adaptive and maladaptive (Stanisławski 2019) depending on how they impact the maintenance of the individual's mental health through stress management and adaptation to adverse circumstances in life (Dias and Pais‐Ribeiro 2019).

Studying the pattern of coping strategies used by the population is important for elucidating how they can relate to stress reduction and ensuring the well‐being of individuals (Tao et al. 2024). This knowledge becomes even more relevant when applied to the university context. Several studies (Campos et al. 2021; Deasy et al. 2014; Graner and Cerqueira 2019) have shown that significant levels of stress and subjective distress have been found in higher education students. This population group may be more likely to develop symptoms of depression, anxiety, and subjective distress (Auerbach et al. 2018; Evans et al. 2018) due to emerging adulthood, which represents a time marked by the search for an identity when a stable life structure has not yet been established. During this period, students seek to understand their place in a world surrounded by instability and are faced with many possibilities, such as career choices, romantic relationships, and gender identity (Gobbo and Dellazzana‐Zanon 2023). This instability can contribute to reduced social support and increased stress, which in turn affect mental health (Slavich and Auerbach 2018). Considering that university students are a risk group for developing symptoms of mental disorders (Batra et al. 2021; Fragelli and Fragelli 2021), it is pertinent to track the different coping strategies they use. Identifying them from a cross‐national comparison between university students from different countries can help to clarify how different contexts and sociocultural issues impact the pattern of strategies used.

Also, studying the coping patterns of individuals from different cultures may be relevant since their worldview and the way they deal with problems and challenges may be different (Chun et al. 2006). Different stressors are expected, as well as the differentiated use of strategies considering the influence of the environment on the individual and the meaning and relevance attached to them. There are cultural variables that must be considered when studying how students have faced challenges, especially when the educational system of each country is structured in completely different ways.

Finland is currently considered, for the eighth consecutive year, the “happiest country in the world” according to the World Happiness Report (WHR) prepared by the UN Sustainable Development Solutions Network (Helliwell et al. 2025). This result is based on variables such as GDP per capita, life expectancy, social support, and freedom to make choices, as assessed by citizens considering the period of the last 3 years. In addition, its education system is essentially public and has policies that prioritize education and value teachers with an integrated and systemic perspective to prepare students for life's challenges (Sahlberg 2018).

In contrast, Brazil has the population with the highest prevalence of anxiety disorders in the world, according to the World Health Organization (Carvalho 2023; Davison et al. 2021). Furthermore, it has an educational system that reflects the social inequalities faced by the population in accessing technologies and information (Pereira et al. 2020). For those with financial stability, access to quality education and other services and basic needs is facilitated, unlike the reality experienced by those with a low monthly income that is not compatible with the cost of living in the country (Trezzi 2022).

Studies conducted in Finland with university students (Bhurtun et al. 2021; Jääskeläinen et al. 2022) noted that, in general, students use strategies considered maladaptive, such as “Self‐distraction” and “Behavioral Disengagement,” when they experience high levels of stress or feel pressured by academic demands, tending to avoid and omit the problem. The use of strategies considered adaptive and centered on emotion, such as “Instrumental Support” and “Emotional Support,” showed a negative correlation with stress and feelings of overload. For Brazilian university students, other studies found similar results (Fonseca and Soares 2023; Hirsch et al. 2015; Santos and Silva 2022). The more difficulties students face, the greater the use of strategies such as “'Denial” and “Self‐distraction” as a way of suppressing emotions and reactions to obtain some kind of control. The use of adaptive and problem‐focused strategies, such as “Planning” and “Active Coping”, correlated positively with the academic adaptation process and problem‐solving ability (Sahão and Kienen 2021). While these studies offer information about coping strategies among university students in Brazil and Finland, they were conducted separately and did not involve a direct cross‐national comparison. As a result, they fall short of helping to understand how sociocultural context might influence similarities or differences in coping behaviors between university students from both countries.

Individual characteristics may play a role in the adoption of coping strategies. Younger people used more maladaptive strategies compared to older people (Hanfstingl et al. 2023). Regarding the variable sex, the literature has suggested that females have greater use of emotion‐focused coping strategies (“Emotional support” and “Venting of emotions”) than males (Panayiotou et al. 2021). Engaging in behaviors that support mental health has also been identified as an individual factor associated with the use of more adaptive coping strategies (Brehl et al. 2021; Budimir et al. 2021). Therefore, considering individual characteristics when studying coping strategies can help identify possible groups that are more vulnerable in each country to the use of adaptive or maladaptive strategies, providing an important perspective for the development of targeted actions and interventions aimed at promoting mental health.

Some studies have also verified how coping strategies relate to the perceived stress in adult populations (Campos et al. 2024; Hanfstingl et al. 2023; Kołodziejczyk et al. 2021; Scorsolini‐Comin et al. 2021). A positive correlation was observed between the use of coping strategies considered maladaptive (“Self‐Blame” and “Behavioral Disengagement”) and the manifestation of symptoms of anxiety, depression, insomnia, psychological distress, and stress. These studies also pointed out that coping strategies more adapted to the context, such as “Active Coping,” “Planning,” “Instrumental support” and “Positive reframing” can act as a protective factor in the face of these symptoms. Given that individuals often use multiple coping strategies simultaneously (Stanisławski 2019), it becomes important to investigate how these strategies interact with one another. Understanding the interconnections may help identify which strategies play a central role in shaping coping patterns and how they are related to students' well‐being.

So far, the only study that has used the BriefCOPE Inventory with a cross‐national approach in university students was conducted by Mojaverian et al. (2012). The study investigated cultural differences between Americans and Japanese students in professional help seeking, focusing specifically on the “'Instrumental Support” and “Emotional Support” strategies. The results showed that Japanese students reported greater reluctance to seek professional help compared to Americans. This is the first study to investigate coping strategies for Brazilian and Finnish university students with a cross‐national approach to compare the results. This comparison may identify differences and similarities between the countries, which can contribute to a broader understanding of identities and experiences from a cultural perspective that can elucidate, above all, the daily challenges faced by these students and how they deal with them. It will also provide important information about contexts that favor the well‐being and mental health of university students. The results of this study may offer indicators associated with cultural differences that may be relevant for the academic community in structuring higher education that is concerned with increasing and strengthening students' socio‐emotional skills and repertoire to better manage everyday psychosocial demands.

Therefore, the objectives of this study are to (i) estimate the psychometric properties of the BriefCOPE inventory in samples of Brazilian and Finnish university students, (ii) conduct a cross‐national comparison (Brazil and Finland) of coping strategies, (iii) identify the relationship between individual characteristics and coping strategies, and (iv) identify the interconnections among the coping strategies within each country.

## 2 Methods

### 2.1 Study Design and Ethical Aspects

**FIGURE 1:**
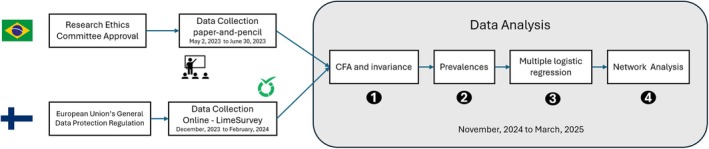
Stages of the research procedure. In “Data Analysis” step 1–4 were related to achieving objectives i, ii, iii, and iv, respectively.

This is a cross‐sectional observational study with a non‐probabilistic sampling design (snowball method) which followed the STROBE reporting guideline. In Brazil, data collection was carried out in 2023 from May 2 to June 30. Students enrolled in the undergraduate courses in Pharmacy and Bioprocess Engineering and Biotechnology at the School of Pharmaceutical Sciences (UNESP–Araraquara) participated in the study by completing the questionnaires using paper‐and‐pencil. These courses were chosen for convenience, since this is the researchers' institution. Initially, the professors were asked to set aside a space in their classroom to administer the questionnaires during regular activities. After this authorization, the students were invited to participate in the research if they agreed and signed the informed consent form. This study is part of a larger project whose implementation was approved by the National Research Ethics Committee of the Ministry of Health (CONEP) (CAAE 30604220.4.0000.0008). In Finland, data were collected throughout 2023 and 2024. Students enrolled at the University of Tampere, University of Turku, and the University of Eastern Finland were invited to participate in the study. The survey was completed exclusively online, generated using Lime Survey, and distributed according to the instructions of the Data Protection Officer at Tampere University, following the European Union's General Data Protection Regulation (Figure 1).

The minimum sample size was estimated using *α* = 5%, *ε* = 15%, and estimates of the mean scores of the different coping strategies adopted by students at the School of Pharmaceutical Sciences at UNESP obtained in previous studies carried out by the research group of the authors of this text (Campos et al. 2021). Considering the coping strategy with the greatest variability, “Substance Use” (mean score = 0.671, standard deviation = 1.041), these estimates were used to define the sample size. Thus, the minimum size for each sample was estimated at 153 participants.

### 2.2 Sample Characterization

To characterize the samples, information such as sex (female, male, I prefer not to inform, other), age (years), monthly family income (Brazil: from 0 to R$1254.00, R$1255.00 to R$2004.00, R$2005.00 to R$8640.00, R$8641.00 to R$11,261.00 and above R$11,261.00; Finland: from 0 to €833, €834 to €1666, €1667 to €2499, €2500 to €3333 and above €3333). Additionally, students were asked whether they take care of their mental health (yes, no). Those who answered “yes” were further asked what actions they consider part of maintaining their mental health (I take medication, I go to therapy, I pay attention to my lifestyle, others).

### 2.3 Measuring Scale

The coping strategies used by the students were identified using the BriefCOPE Inventory (Carver 1997). It is a self‐report scale consisting of 28 items grouped into 14 factors (Active Coping—AC, Planning—PL, Instrumental Support—IS, Emotional Support—ES, Religion—RE, Positive Reframing—PR, Self‐blame—SB, Acceptance—AT, Venting of Emotions—VE, Denial—DN, Self‐distraction—SD, Behavioral Disengagement—BD, Substance Use—SU, and Humor—HU). The Portuguese version was used in this study for the Brazilian sample (Marôco et al. 2014), while the Finnish version was used for the Finnish sample (Mikkola 2013). The response scale is a 5‐point Likert‐type format (0 = *I've never done this*; 1 = *I've done this before*; 2 = *Sometimes*; 3 = *I usually do this*; and 4 = *I always do this*). For statistical analyses, the mean scores for each BriefCOPE factor were calculated. These scores were divided into two categories: commonly used strategies (score ≥ 3) and rarely used strategies (score < 3) (Campos et al. 2022).

From a theoretical perspective, the 14 factors can be grouped into adaptive (e.g., AC, PL, IS, ES, RE, PR, AT, VE, HU) or maladaptive (e.g., SB, DN, SD, BD, SU) strategies, as well as can be divided into emotion‐focused (e.g., IS, ES, VE, HU) or problem‐focused (e.g., AC, PL, PR, AT, RE) strategies (Stanisławski 2019). However, this grouping strategy is not consensual and may vary among authors and studies depending on their context of use. Despite this, we relied on this classification to interpret our results, since we understand that it is appropriate for the type of context in which BriefCOPE was applied.

### 2.4 Data Validity and Reliability

The validity and reliability of the data obtained with the BriefCOPE Inventory were estimated by confirmatory factor analysis (CFA). Initially, the psychometric sensitivity (Marôco 2021) of the items was assessed to check if they presented normal distribution (Skewness ≤ |3|; kurtosis ≤ |10|). The fit of the BriefCOPE factor model to the samples was then estimated with a robust weighted least squares method adjusted for mean and variance (WLSMV) and using the Comparative Fit Index (CFI), Tucker‐Lewis Index (TLI), and Root Mean Square Error of Approximation (RMSEA) indices with a 90% confidence interval. The fit of the factor model was considered adequate when CFI and TLI ≥ 0.90 and RMSEA ≤ 0.10 (Hair et al. 2019; Kline 2016; Marôco et al. 2014). The factor loading (*λ*) of the items was evaluated and considered satisfactory when *λ* ≥ 0.50 (Hair et al. 2019). Reliability and internal consistency were estimated based on the omega coefficient (*ω*), ordinal alpha coefficient (*α*), and Cronbach's alpha. Values of *ω*, *α*, and Cronbach's alpha ≥ 0.70 were considered adequate. For these analyses, R (Team 2016) was used with the packages “lavaan” (Rossel 2012) and “semTools” (Jorgensen et al. 2018).

### 2.5 Data Analysis

For all analyses, the categories “prefer not to inform” and “other” of the sex variable were disregarded, as they did not have enough participants to perform statistical inference. For the individual characteristics from each sample the prevalences were calculated and a chi‐square test (*α* = 5%) was performed between them. The prevalence of commonly used coping strategies (mean scores ≥ 3) was calculated for each sample using a 95% confidence interval (95% CI). Multiple logistic regression models were developed for each sample and the odds ratio (OR) estimated (95% CI) to identify the probability of adopting each of the different coping strategies (dependent variable) based on sample characteristics. A Network analysis was conducted to verify the relationship between the different coping strategies used by students and to target the main strategy maintaining the students' coping pattern in each country (Leme et al. 2020). A Gaussian graphical model (Borsboom et al. 2021) regulated by the Least Absolute Shrinkage and Selection Operator (LASSO) method was used. The standardized node strength, closeness, and betweenness centrality indices were estimated and inspected (Epskamp et al. 2018). The analyses were performed using the JASP Team (2021) version 0.16 program.

## 3 Results

The study included 398 Brazilian students and 165 Finnish students. Table 1 presents the characteristics of the samples. In both samples, most participants were female. The mean age for the Brazilian sample was 21.0 years (SD = 2.2) and 26.9 years (SD = 7.2) for the Finnish sample. In Brazil, the most prevalent category for monthly income was R$2005.00 to R$8640.00, while in Finland, it was the lowest category, up to €833. In both samples, most students reported taking care of their mental health. The most reported type of care was “paying attention to lifestyle.”

**TABLE 1:** Sample characterization, *n* (%).

Characteristic		Brazil, *n* = 398	Finland, *n* = 165	*X* ^2^ (*p*)	Effect size^a^
Sex					
Male		124 (31.2)	45 (27.3)		
Female		265 (66.6)	111 (67.3)		
Prefer not to inform		2 (0.5)	4 (2.4)		
Other		7 (1.7)	5 (3.0)	6.619 (0.085)	0.108
Monthly family income^b^					
Brazil (R$)	Finland (€)				
≤ 1254.00	0–833	10 (2.5)	72 (43.6)		
1255.00–2004.00	834–1666	53 (13.4)	38 (23.0)		
2005.00–8640.00	1667–2499	228 (57.6)	13 (8.0)		
8641.00–11261.00	2500–3333	57 (14.4)	9 (5.5)		
≥ 11262.00	≥ 3334	48 (12.1)	33 (19.9)		
Mental health care					
No		91 (22.9)	23 (13.9)		
Yes		307 (77.1)	142 (86.1)	5.214 (0.022)	0.101
Type of mental health care^c^					
I use medication		60 (15.1)	24 (16.9)	0.145 (0.704)	0.022
I go to therapy		89 (22.4)	24 (16.9)	1.570 (0.210)	0.059
I pay attention to my lifestyle		287 (72.1)	126 (88.7)	15.165 (< 0.001)	0.173
Other		18 (4.5)	24 (16.9)	52.770 (< 0.001)	0.597

*Note:* Data collection. Brazil: May 2 to June 30, 2023; Finland: throughout the years 2023 and 2024.

^a^ Phi coefficient or Cramer's *V*.

^b^ The categories of monthly family income do not coincide between countries, so the chi‐squared test was not performed for this variable.

^c^ More than one category could be selected by the participant.

Initially, the psychometric sensitivity of the items was analyzed using descriptive statistics of the responses given to them by the students. All items presented responses without severe violation of the normal distribution in each sample (skewness ≤ |2|; kurtosis ≤ |10|) (Kline 2016; Marôco 2021). Therefore, psychometric sensitivity was confirmed, which supported the subsequent conduct of confirmatory factor analysis.

The complete BriefCOPE model did not present an adequate fit to the data in any of the samples. For the Brazilian sample, items that compose the SU factor (bc25 and bc26) presented a correlation value close to 1, indicating possible redundancy, and items bc14 (SB factor), bc26 (SU factor) and bc27 (HU factor) presented negative variance. After the exclusion of those factors, the model still did not present an adequate fit to the data, and item bc10 (RE factor) had a negative variance value. For the refinement of the model, the RE factor was also excluded. The final refined model with 10 factors presented adequate validity and reliability of the data (Table 2).

**TABLE 2:** Psychometric indicators of the factorial models of the BriefCOPE Inventory fit to the samples.

			Confirmatory factor analysis							
Sample	Model	Excluded factors	*λ*	CFI	TLI	RMSEA [IC 90%]	*ω*	*α*	Cronbach's alpha [IC 95%]	Observation
Brazil	Complete	—		—	—	—	—	—	*—*	The covariance matrix of the factor model was not defined as positive: items of the SU factor with correlation close to 1 and items bc14 (SB), bc26 (SU) and bc27 (HU) with negative variance.
	11 Factors	SB, SU, and HU	—	—	—	—	—	—	—	Item BC10 (RE) with negative variance.
	10 Factors	RE, SB, SU, and HU	0.77–0.97	0.962	0.943	0.071 [0.058 —0.083]	0.77–0.92	0.82–0.95	0.078 [0.074–0.081]	
Finland	Complete	—	—	—	—	—	—	—	*—*	The covariance matrix of the factor model was not defined as positive: items of the RE, DN, BD and SU factors without adequate psychometric sensitivity
	10 Factors	RE, DN, BD, and SU	*—*	*—*	*—*	*—*	*—*	*—*	*—*	The covariance matrix of the factor model was not defined as positive: items bc11 (PR) and bc16 (AT) with negative variance
	8 Factors	RE, PR, AT, DN, BD, and SU	0.61–0.99	0.922	0.876	0.109 [0.085–0.133]	0.71–0.91	0.72–0.95	0.086 [0.083–0.089]	

*Note:* *λ*, Factor loading; CFI, Comparative Fit Index; TLI, Tucker‐Lewis Index; RMSEA, Root Mean Square Error of Approximation; *ω*, omega coefficient; α, ordinal alpha coefficient; AT, acceptance; BD, behavioral disengagement; DN, denial; RE, factors: religion; HU, humor; PR, positive reframing; SB, self‐blame; SU, substance use.

For the Finnish sample, the RE, DN, BD, and SU factors showed insufficient variability in the responses given to their items, with a greater propensity for students to respond to the lowest value categories (0 = I've never done this; 1 = I've done this before; 2 = Sometimes). Even after excluding the above factors, the model did not show adequate fit, and items bc11 (PR factor) and bc16 (AT factor) showed negative variance; therefore, the model was refined again by excluding the PR and AT factors. This final model for the Finnish sample showed adequate validity and reliability of the data (Table 2).

These results indicate a lack of configural invariance between samples, which makes it impossible to make direct comparisons of the coping strategies used by students from the different countries (Cheung and Rensvold 2002). For the statistical analyses, the final models obtained for each sample (Brazil and Finland) were used separately.

Table 3 shows the prevalence of commonly used coping strategies (mean scores ≥ 3) by Brazilian and Finnish students. In Brazil, the most prevalent strategies were “Planning” and “Self‐distraction,” and in Finland, they were “Emotional Support” and “Venting of Emotions”. In general, for the Finnish sample, all strategies presented considerable and more evenly distributed prevalence values, the opposite of what is observed for the Brazilian sample.

**TABLE 3:** Prevalence (p, 95% CI) of coping strategies commonly used (mean scores ≥ 3) by students.

BriefCOPE	Brazil		Finland	
	*p* (%)	IC95%	*p* (%)	IC95%
Active Coping (AC)	44.2	39.2–49.1	22.4	15.8–29.0
Planning (PL)	55.3	50.4–60.2	31.4	24.1–38.7
Instrumental Support (IS)	39.6	34.7–44.5	29.5	22.3–36.7
Emotional Support (ES)	44.7	39.8–49.6	41.0	33.2–48.7
Positive Reframimg (PR)	25.7	21.4–30.0	—	—
Self‐blame (SB)	—	—	23.7	17.0–30.4
Acceptance (AT)	32.6	27.9–37.3	—	—
Venting of emotions (VE)	32.4	27.7–37.0	33.3	25.9–40.7
Denial (DN)	4.9	2.8–7.0	—	—
Self‐distraction (SD)	50.6	45.6–55.6	24.4	17.6–31.2
Behavioral Disengagement (BD)	6.9	4.4–9.4	—	—
Humor (HU)	—	—	31.4	24.1–38.7

*Note:* Excluded factors from analyses due to the refined factorial model. Brazil: RE (Religion), SB (Self‐blame), SU (Substance Use) and HU (Humor); Finland: RE (Religion), PR (Positive Reframing), AT (Acceptance), DN (Denial), BD (Behavioral Disengagement) and SU (Substance use).

Table 4 presents the probability of using each of the coping strategies considering the individual characteristics for each sample (Brazil and Finland). For the Brazilian sample, mental health care was a relevant characteristic for increasing or decreasing the probability of using different coping strategies by students. As for the Finnish sample, individual characteristics seem to have little effect on the choice of coping strategies. In both samples, female students were more likely to use emotion‐focused coping strategies, such as “Emotional Support” and “Venting of emotions”. In Brazil, for students who report having some type of mental health care, there is a greater probability of using adaptive strategies, whether they are emotion‐ or problem‐focused. The opposite is found for Brazilian students without any care, who were more likely to use maladaptive strategies, such as “Behavioral Disengagement.”

**TABLE 4:** Odds Ratio (OR, 95% CI) of coping strategies (< 3 = rarely used/≥ 3 = commonly used) according to sample characteristics.

Sample	Strategy	Characteristics					
		Sex (male^a^)	Monthly family income (≥ R$11,262.00^a^), < R$1255.00	R$ 1255.00–R$2004.00	R$ 2005.00–R$8640.00	R$ 8640.00–R$11,261.00	Mental health care present/absent^a^
Brazil	AC	1.42 (0.90–2.24)	2.08 (0.48–8.94)	0.90 (0.39–2.05)	0.84 (0.43–1.62)	0.68 (0.30–1.56)	3.51 (2.01–6.14)*
	PL	0.94 (0.60–1.48)	0.76 (0.17–3.30)	0.28 (0.12–0.67)*	0.56 (0.27–1.14)	0.32 (0.14–0.75)*	2.82 (1.70–4.68)*
	IS	1.93 (1.21–3.08)*	1.02 (0.24–4.23)	1.59 (0.70–3.65)	0.88 (0.45–1.72)	0.76 (0.33–1.74)	1.92 (1.13–3.26)*
	ES	3.12 (1.93–5.05)*	0.47 (0.11–1.99)	0.58 (0.25–1.37)	0.50 (0.26–1.00)	0.53 (0.23–1.21)	2.13 (1.26–3.60)*
	PR	0.77 (0.47–1.26)	0.83 (0.19–3.70)	0.82 (0.35–1.92)	0.60 (0.30–1.16)	0.26 (0.10–0.71)*	1.29 (0.72–2.31)
	AT	0.81 (0.51–1.28)	1.17 (0.28–4.79)	0.65 (0.27–1.54)	0.78 (0.40–1.52)	1.13 (0.50–2.55)	0.86 (0.52–1.43)
	VE	2.15 (1.30–3.56)*	0.94 (0.23–3.92)	0.87 (0.38–2.00)	0.55 (0.28–1.10)	0.67 (0.29–1.54)	1.58 (0.91–2.74)
	DN	2.44 (0.69–8.58)	—	2.56 (0.25–25.94)	2.34 (0.29–18.65)	2.47 (0.24–24.92)	2.55 (0.57–11.36)
	SD	1.34 (0.87–2.08)	3.35 (0.63–17.67)	0.68 (0.30–1.52)	0.80 (0.42–1.51)	1.33 (0.60–2.95)	0.94 (0.58–1.53)
	BD	1.39 (0.56–3.44)	—	1.12 (0.18–7.21)	1.56 (0.34–7.14)	2.18 (0.41–11.70)	0.39 (0.17–0.88)*

Sample	Strategy	Characteristics					
		Sex (male^a^)	Monthly family income (≥ €3334^a^) < €834	€834–€1666	€1667–€2499	€2500–€3333	Mental health care Present/Absent^a^
Finland	AC	1.02 (0.40–2.57)	0.39 (0.15–1.05)	0.19 (0.05–0.67)	0.96 (0.24–3.91)	0.19 (0.02–1.73)	6.94 (0.86–56.02)
	PL	0.62 (0.28–1.37)	0.34 (0.14–0.87)*	0.29 (0.10–0.84)*	0.88 (0.22–3.46)	0.52 (0.11–2.50)	2.34 (0.70–7.78)
	IS	0.98 (0.43–2.24)	0.88 (0.35–2.23)	0.35 (0.11–1.12)	1.59 (0.40–6.33)	0.51 (0.09–2.94)	4.52 (0.98–20.82)
	ES	3.29 (1.37–7.93)*	1.83 (0.72–4.63)	0.64 (0.22–1.87)	13.56 (2.26–81.48)*	4.44 (0.87–22.55)	2.59 (0.77–8.73)
	SB	0.82 (0.36–1.88)	1.33 (0.46–3.86)	1.80 (0.58–5.61)	1.36 (0.27–6.82)	0.53 (0.06–5.12)	1.04 (0.34–3.17)
	VE	1.63 (0.72–3.69)	0.61 (0.25–1.49)	0.42 (0.15–1.16)	0.57 (0.14–2.35)	0.14 (0.02–1.25)	2.27 (0.70–7.42)
	SD	0.88 (0.38–2.03)	1.36 (0.46–3.97)	1.62 (0.51–5.16)	4.72 (1.08–20.69)*	—	0.53 (0.18–1.50)
	HU	0.60 (0.28–1.29)	1.16 (0.44–3.10)	1.74 (0.61–4.95)	0.80 (0.17–3.82)	2.28 (0.48–10.79)	1.79 (0.59–5.40)

*Note:* Coping strategies: AC (Active Coping), PL (Planning), IS (Instrumental Support), ES (Emotional Support), SB (Self‐Blame), PR (Positive Reframing), AT (Acceptance), VE (Venting of Emotions), DN (Denial), SD (Self‐Distraction), BD (Behavioral Disengagement) and HU (Humor). Excluded factors from the analyses due to the refined factorial model. Brazil: RE, SB, SU, and HU; Finland: RE, PR, AT, DN, BD, and SU.

^a^ rc, reference category.

* *p* < 0.05.

Table 5 presents the network analyses performed for each sample. When analyzing the indices of the Brazilian sample, “Planning” played an important role in determining the correlations (node strength) with other nodes (coping strategies), while “Behavioral Disengagement” was an important mediator between the other strategies (node betweenness) and in determining their indirect connections (node closeness). For the Finnish sample, in addition to being the central node of the network, “Planning” is the main strategy that determines the correlations and indirect connections with other nodes and plays an important role as an intermediary between other pairs of strategies. The accuracy and stability of the networks were estimated and attested through nonparametric bootstrapping and case‐dropping, respectively, and the results can be found in Supporting Information.

**TABLE 5:** Network analysis: Centrality indices node strength, closeness, and betweenness for each sample.

Sample	Brazil (*n* = 389)				Finland (*n* = 156)			
	Centrality indices				Centrality indices			
Strategy	Betweenness	Closeness	Strength	Expected influence	Betweenness	Closeness	Strength	Expected influence
AC	0.471	0.781	0.636	0.881	−1.183	−0.363	−0.023	−0.023
PL	0.994	1.058	1.520	1.023	1.466	1.191	1.515	1.515
IS	0.209	0.165	0.836	1.222	0.710	1.022	0.968	0.968
ES	0.209	0.165	1.166	1.213	−0.047	0.390	0.547	0.547
PR	−1.360	−0.506	−0.306	−0.785	—	—	—	—
SB	—	—	—	—	1.088	0.003	−0.436	−0.436
AT	−1.360	−2.012	−1.229	−0.684				
VE	0.209	−0.203	−0.432	−0.253	−0.426	0.319	−0.051	−0.051
DN	0.732	0.611	−0.664	−0.259				
SD	−1.360	−1.147	−1.408	−0.848	−1.183	−1.940	−1.034	−1.034
BD	1.255	1.088	−0.120	−1.510				
HU	—	—	—	—	−0.426	−0.622	−1.486	−1.486
Sparsity				0.422				0.286

*Note:* Highlighted boxes represent the main indicators. Excluded factors from the analyses due to the refined factorial model. Brazil: RE, SB, SU, and HU; Finland: RE, PR, AT, DN, BD, and SU.

## 4 Discussion

To our knowledge, this is the first cross‐national study conducted to investigate coping strategies for Brazilian and Finnish university students. Given the situational and dynamic nature of coping strategy patterns according to the theoretical framework proposed by Folkman and Lazarus (1980), sociocultural issues are a relevant aspect for the adoption of different coping strategies. The present study highlights the importance of understanding indicators associated with cultural differences between countries that can elucidate the daily challenges faced and how these students deal with them.

### 4.1 Data Validity, Reliability and Cross‐national Invariance

The validity and reliability of the data obtained with BriefCOPE attest to the quality of the results found. In the preliminary phase, some refinements in the BriefCOPE factor model were necessary for an adequate fit to the data. For both samples (Brazil and Finland), it was necessary to exclude the factors “Substance Use” and “Religion”. These factors had insufficient variability, with a greater propensity for students to respond to the smallest response scale options (0 = I've never done this; 1 = I've done this before). While in Finland this scenario could be explained by a declining trend in substance use among higher education students (Raitasalo et al. 2024), in Brazil, this is not seen to be the reality indicated in the literature (Barbosa et al. 2020; Oliveira et al. 2013). One possible interpretation for that result among Brazilian students is associated with social desirability, so that they may have responded to the items according to behaviors that are expected and socially accepted, but that do not necessarily correspond to their behavior (Almiro 2017; Persson et al. 2025). As for the “Religion” factor, its exclusion suggests a movement of religious disengagement among the younger generations (Räsänen 2010). Above all, emerging adulthood is marked by wonderings about beliefs and values established during childhood, so that it is a period in which young people generally distance themselves from religion and religious practices (Denton et al. 2008; Niemelä 2015).

Furthermore, we observed a lack of configural invariance of the BriefCOPE factor model between the countries, which points to major cultural differences in the pattern of coping strategies. For the Brazilian sample, in addition to “Substance Use”, it was also necessary to exclude the factors “Humor” and “Self‐blame”. One possible interpretation for this may be the Brazilian reality sustained by many social inequalities, so it is difficult for people, in general, to use humor to solve or face everyday challenges that are often beyond their control. In the Brazilian reality and context, many of the challenges that are imposed on the population daily are perceived as the responsibility of the current government, its public policies, and structural socioeconomic issues (Carvalho et al. 2021; Pitombeira and Oliveira 2020). For the Finnish sample, the strategies “Positive Reframing,” “Acceptance,” “Denial,” and “Behavioral Disengagement” were excluded. These results may suggest a non‐conformist and more practical and realistic profile for solving problems and facing challenges (Stanisławski 2019).

### 4.2 Prevalences of Coping Strategies in Finnish and Brazilian Students

The prevalence calculated for coping strategies indicates that Finnish students had a more proportional and diversified distribution of coping strategies. In contrast, the Brazilian sample presented greater variation between the prevalence rates, with emphasis on the use of the strategies “Planning” and “Self‐distraction”. These strategies are considered antagonistic: while “Planning” is an adaptive strategy focused on the problem, “Self‐distraction” can be considered maladaptive, with a tendency for the subject to avoid thinking about the problem, postpone decision‐making, and reduce efforts to solve it (Agha 2021; Campos et al. 2022; Campos et al. 2020). These results indicate that Brazilian students, when faced with challenges and stressful situations, opt for two extremes: either they actively try to solve their problems or passively choose to ignore them. Finnish students, on the other hand, appear to have a greater ability to mobilize coping strategies, which implies better management between expectations and reality and better psychosocial adaptation that is positively related to the maintenance of physical and mental health and quality of life (Brehl et al. 2021; Lazarus and Folkman 1984).

### 4.3 Sample Characteristics and Coping Strategies

When considering individual characteristics, it was possible to identify important cultural differences in establishing the pattern of coping strategies. In the Finnish sample, students with a higher‐level income are more likely to use adaptive strategies such as “Planning”, which perhaps indicates that the low‐income level of most students can be a major stress in their lives (Jääskeläinen 2022). However, compared to the Brazilian sample, these individual characteristics seem to be less significant for the adoption of different strategies. As Finland is a more egalitarian country, students may have a coping repertoire based on the facilitated access they have to health and education, which implies less exposure to risk situations. Consequently, everyday challenges may be more attenuated than those presented and/or perceived by Brazilian students, so Finnish students were able to build a more diversified coping pattern with the use of more effective and appropriate strategies for managing problems, since social and/or political issues may not probably affect their daily lives as directly as they do for Brazilians (Waltenberg and Martins 2021).

For the Brazilian sample, students who reported having some type of mental health care were more likely to use adaptive strategies, such as “Active Coping,” “Planning,” “Instrumental Support,” and “Emotional Support,” while the lack of care contributed to the increased chances of using maladaptive strategies, such as “Behavioral Disengagement.” Taking care of mental health is a protective factor and contributes to the use of more adaptive coping strategies, while the use of maladaptive strategies has been associated with higher levels of stress, anxiety, lower well‐being, and interpersonal functioning, which can lead to mental health impairment (Brehl et al. 2021; Budimir et al. 2021). Therefore, these results reinforce the importance of promoting mental health and a healthy lifestyle among young people through spaces that allow, for example, physical exercise, access to a nutritious diet, and healthy ways of managing stress (Çetinkaya and Sert 2021; Vieira et al. 2021).

### 4.4 Connection Between Coping Strategies: Network Analysis

The most relevant coping strategies and their connections identified in the network analysis indicate that, in general, “Planning” is the central strategy in problem‐solving for students in both countries. This strategy focuses on the problem and involves recognizing it in order to seek to solve it by taking action and trying to eliminate the stressor (Stanisławski 2019; Steinhardt and Dolbier 2008). Interestingly, in addition to its central role, the “Planning” strategy in the Finnish sample was also relevant for establishing the coping pattern, and mediating the relationship between the other strategies, which reinforces a more problem‐solving profile in this population. In the Brazilian sample, “Behavioral disengagement” proved to be relevant in establishing the students' coping pattern. It should be emphasized that both “Self‐distraction”, one of the most prevalent strategies for the Brazilian sample, and “Behavioral Disengagement” compose a dimension called helplessness (Stanisławski 2019), which combines avoidance and negative emotions, indicating that Brazilian students have a smaller repertoire of solutions, which tends toward escapism and negativism. This may be related to, for example, the economic and social inequalities already reported.

### 4.5 Cross‐National Comparison

Given the lack of configural invariance of the BriefCOPE between Finland and Brazil, a direct comparison of the results is not possible. Nevertheless, it seems worthwhile to formulate hypotheses in an attempt to understand the factors contributing to the divergent operationalization of the instrument across the countries.

Overall, the results found for the Finnish sample seem to reflect the impact of a high‐standard educational system that understands development as freedom and that presents high indicators of students' quality of life (Waltenberg and Martins 2021). Finland is a country that prioritizes investment in education in an equitable manner and in broader learning. Among the principles that guide its education is the development of individuals capable of dealing with challenges in an effective, creative, and innovative way (OECD 2014). Compared to other countries, Finnish students have reported superior results for the level of satisfaction with life (OECD 2017). This scenario contributes to higher levels of education and can be associated with a profile of greater predisposition to self‐control, health care, and better management of interpersonal relationships (Feinstein et al. 2008). This may partly explain why Finnish students seem to have more interesting coping repertoires.

The results for the Brazilian sample seem to reflect the current scenario of social inequality and mental health care in the country. Brazil has a higher prevalence of common disorders in higher education students compared to the general population (Gomes et al. 2020; Graner and Cerqueira 2019; Lopes et al. 2016). These disorders are characterized by the presence of symptoms of anxiety and depression, associated with intense psychological distress, which impact mental health and, consequently, the pattern of coping (Fisher et al. 2022). These indicators may be related to the use of more maladaptive strategies by Brazilian students. Furthermore, the country has a deficient educational system that is discrepant between social classes (Bertolin and McCowan 2022; Rossi et al. 2019), which plays a determining role in increasing inequalities in access to quality education (Medeiros et al. 2020). Added to a teaching–learning process compromised by socioeconomic issues, it is possible that students will have difficulty developing more adaptive coping repertoires that are appropriate for everyday challenges.

This study was conducted with the aim of raising awareness about sociocultural differences and their impact on the pattern of use of coping strategies by Brazilian and Finnish university students. We hope that the results can provide indicators that may be relevant for establishing educational, preventive, and intervention measures that strengthen the socioemotional repertoire and skills of university students to better manage the daily psychosocial demands specific to this population.

### 4.6 Limitations and Future Perspective

This study has some limitations. First, the use of a non‐probabilistic sampling prevents generalization of the results to the entire population, as the sample may not be fully representative of university students in Brazil or Finland. Second, as this is a cross‐sectional observational study, causal inferences cannot be made (Hernán and Robins 2020), which limits the possibility of determining the directionality of the relationships between coping strategies and individual characteristics. Third, the Finnish sample size was smaller than the Brazilian one. BriefCOPE Inventory has 28 items, which generally requires a larger sample for the fit of the factorial model (Bonett 2002; MacCallum et al. 2001). Additionally, other relevant factors that can contribute to the pattern of coping strategies, such as personality and previous diagnosis of mental disorders, were not considered in the present study, despite their well‐established relationship in the literature (Aldao et al. 2010; Bolger 1990; Connor‐Smith and Flachsbart 2007). The absence of these variables may have limited the interpretation of the relationships observed between coping strategies and individual characteristics. Therefore, future studies should aim to include larger and more balanced samples across countries, and consider additional individual, psychological, and clinical variables to better understand the adoption of coping strategies in university students.

## 5 Conclusion

The use of different coping strategies is situational and depends on the context in which the student is inserted and the resources he or she has. In general, Finnish students have a more diverse repertoire for coping with everyday problems and better manage psychosocial demands, while Brazilian students opt for extremes, dedicating themselves to solving or avoiding problems. This fact may be related to both the structuring of repertoires and the nature and complexity of the everyday challenges presented, which are very different between countries. Expanding students' coping repertoire combined with increased use of adaptive strategies through programs and actions aimed at education and maintenance of mental health may be important in preventing the development of symptoms associated with mental disorders in response to stress.

## Author Contributions

**Livia Oliveira dos Santos:** conceptualization, data collection and curation, formal analysis (lead), funding acquisition, investigation, writing – original draft preparation (lead). **Lucas Arrais de Campos:** conceptualization, data collection and curation, investigation, methodology, project administration, writing – original draft preparation (supporting), writing – review and editing. **Adrielly dos Santos:** investigation, formal analysis (supporting), writing – review and editing. **Timo Peltomäki:** funding acquisition, investigation, supervision, writing – review and editing. **Tella Lantta:** conceptualization (supporting), methodology (supporting), data collection, investigation, writing – review and editing. **Jaakko Varpula:** conceptualization (supporting), methodology (supporting), data collection, investigation, writing – review and editing. **João Maroco:** methodology, supervision, writing – review and editing. **Juliana Alvares Duarte Bonini Campos:** conceptualization, funding acquisition, methodology, supervision, project administration, writing – original draft preparation (supporting), writing – review and editing.

## Ethics Statement

In Brazil, this study is part of a broader research project approved by the National Research Ethics Committee of the Ministry of Health (CONEP) (CAAE 30604220.4.0000.0008). In Finland, the online survey was administered in compliance with the recommendations of the Data Protection Officer at Tampere University and in accordance with the European Union General Data Protection Regulation (GDPR). In both countries, participation was voluntary, and only students who provided informed consent were included in the study.

## Conflicts of Interest

The authors declare no conflicts of interest.

## Supporting information


**Data S1:** sjop70013‐sup‐0001‐DataS1.docx.

## Data Availability

The datasets generated during and/or analyzed during the current study are available from the corresponding author on reasonable request.
