# Supplementary material for: The Use of Coping Strategies for Everyday Challenges by University Students: Brazil‐Finland Cross‐National Study
Source: Scand J Psychol. 2025 Aug 6;67(1):12–24. doi: 10.1111/sjop.70013 (PMC12790107; doi:10.1111/sjop.70013)
Supplement: Supplementary file 1 — Data S1: sjop70013‐sup‐0001‐DataS1.docx. [file SJOP-67-12-s001.docx]

**Accuracy and Stability: Network analysis**


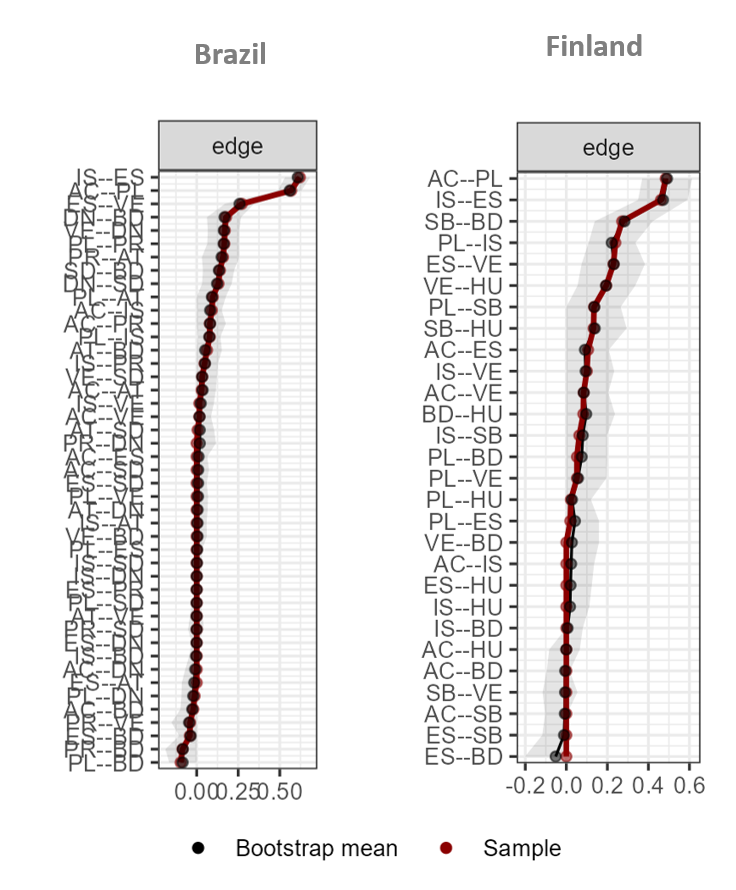


**Figure 1.** Network Analysis: Accuracy of networks for each sample according to mental health care estimated from non-parametric bootstrap.


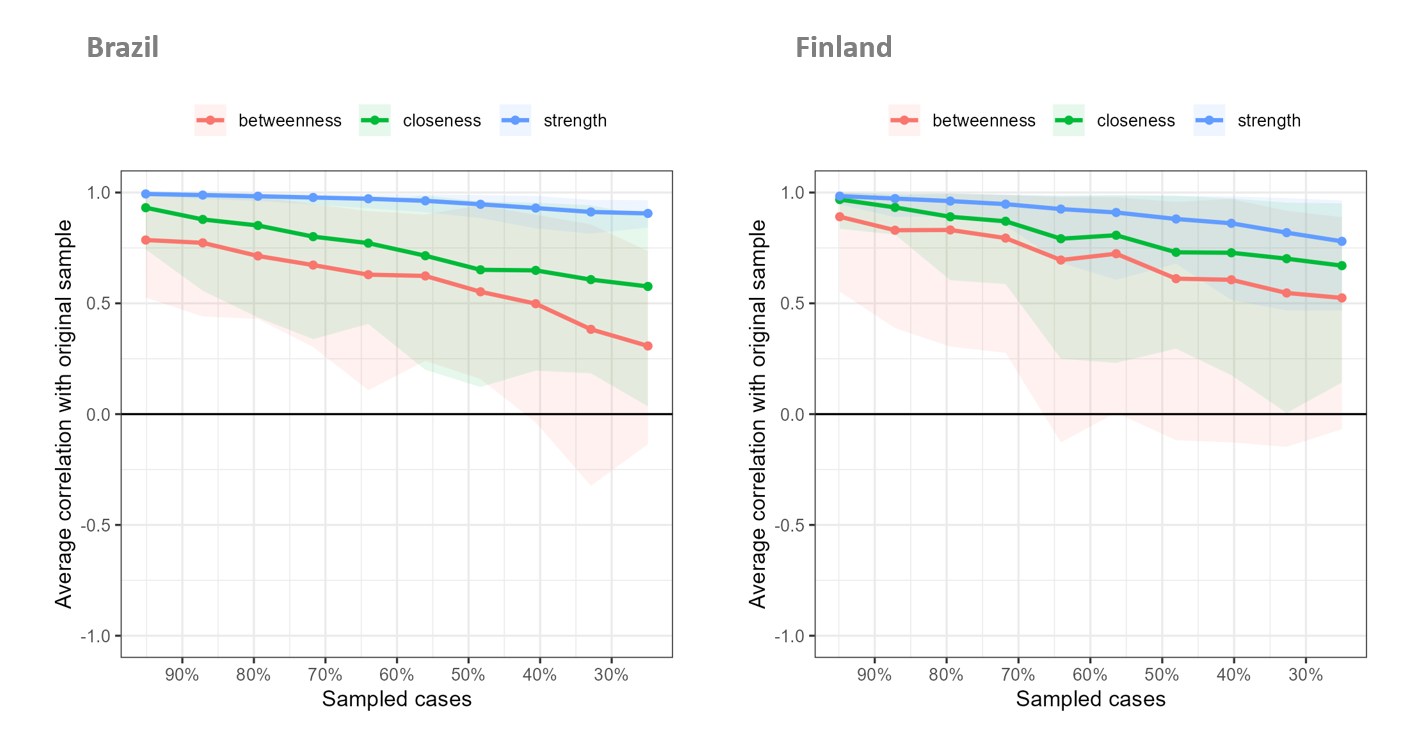


**Figure 2.** Network Analysis: Network stability (Average correlation >0.5) of each sample according to mental health care estimated from case-dropping bootstrap.
